# Effect of Cellulose Nanofiber (CNF) Surface Treatment on Cellular Structures and Mechanical Properties of Polypropylene/CNF Nanocomposite Foams via Core-Back Foam Injection Molding

**DOI:** 10.3390/polym11020249

**Published:** 2019-02-02

**Authors:** Long Wang, Kiyomi Okada, Yuta Hikima, Masahiro Ohshima, Takafumi Sekiguchi, Hiroyuki Yano

**Affiliations:** 1 Department of Chemical Engineering, Kyoto University, Kyoto 615-8510, Japan; okada.kiyomi.6n@kyoto-u.ac.jp (K.O.); hikima@cheme.kyoto-u.ac.jp (Y.H.); 2 New Business Development Division, SEIKO PMC Corp., Chiba 267-0056, Japan; takafumi-sekiguchi@seikopmc.co.jp; 3 Research Institute for Sustainable Humano-sphere, Kyoto University, Kyoto 611-0011, Japan; yano@rish.kyoto-u.ac.jp

**Keywords:** polypropylene, cellulose nanofiber, foam injection molding, mechanical properties

## Abstract

Herein, lightweight nanocomposite foams with expansion ratios ranging from 2–10-fold were fabricated using an isotactic polypropylene (iPP) matrix and cellulose nanofiber (CNF) as the reinforcing agent via core-back foam injection molding (FIM). Both the native and modified CNFs, including the different degrees of substitution (DS) of 0.2 and 0.4, were melt-prepared and used for producing the polypropylene (PP)/CNF composites. Foaming results revealed that the addition of CNF greatly improved the foamability of PP, reaching 2–3 orders of magnitude increases in cell density, in comparison to those of the neat iPP foams. Moreover, tensile test results showed that the incorporation of CNF increased the tensile modulus and yield stress of both solid and 2-fold foamed PP, and a greater reinforcing effect was achieved in composites containing modified CNF. In the compression test, PP/CNF composite foams prepared with a DS of 0.4 exhibited dramatic improvements in mechanical performance for 10-fold foams, in comparison to iPP, with increases in the elastic modulus and collapse stress of PP foams of 486% and 468%, respectively. These results demonstrate that CNF is extraordinarily helpful in enhancing the foamability of PP and reinforcing PP foams, which has importance for the development of lightweight polymer composite foams containing a natural nanofiber.

## 1. Introduction

Polymeric foams have aroused great interest in a variety of fields, including construction, transportation, thermal and sound insulation, together with tissue engineering [1,2,3]. Compared with their solid counterparts, polymeric foams possess many distinctive physical characteristics, such as high impact strength, low density, good energy absorption, and excellent thermal and acoustic insulation [1,2,3]. Typically, polymeric foams can be prepared from different techniques such as extrusion foaming, batch foaming, bead foaming, and foam injection molding. One of the major advantages of the foam injection molding (FIM) technique is that it is feasible to fabricate foam products with complex three-dimensional geometries, whereas this is difficult to achieve with other processing technologies [4]. This makes the FIM process especially appealing in areas such as automotive and electronic packaging. Additionally, in comparison to regular solid injection molding, FIM products exhibit several advantages, such as less material usage, better geometric accuracy, lower energy consumption, and less product shrinkage [4]. To date, extensive work has been reported on preparing polymeric foams using resins such as polyethylene (PE) [5,6], polypropylene (PP) [7,8,9,10], polylactide (PLA) [11,12], and polyamide 6 (PA6) [13] via the FIM technique.

Isotactic polypropylene (iPP) is a widely-used commercial polymeric material and has good overall performance, including easy processing, excellent chemical resistance, high thermal stability, and good mechanical properties [14]. It demonstrates greater strength than other polyolefins such as PE, as well as better impact strength with respect to PS. Moreover, iPP exhibits a higher servicing temperature than PE and PS, which makes iPP more attractive than other thermoplastics in the foam industry. However, the intrinsically weak melt strength of iPP, together with its tendency to crystallize into sizable spherulites make iPP foam suffers from poor cellular structures and inferior mechanical properties, which unavoidably restrict it from extensive applications [8,9,15]. Accordingly, the improvement of the foamability of PP has always been open to further research. Until now, several approaches have been employed to enhance the foaming property of PP, including blending with other polymers [16,17], chemical crosslinking and/or introducing long-chain branching [7,18,19,20], compounding with inorganic or organic particles (e.g., nanofillers) [21,22,23,24], and adding a special nucleating agent [7,8]. There is no doubt that these avenues, to a greater or lesser extent, improve the foamability of iPP and expand its application.

The incorporation of nanoparticles is a simple and viable approach, which not only improves the melt strength of iPP but also promotes its crystallization property. Thus, this would notably improve the cellular structures of iPP and enhance its foaming ability. Usually, nanoparticles, such as nanoclay [22,23], carbon nanotubes [25,26], and carbon nanofibers [27] have been used to improve the foamability of iPP. Additionally, compared with other modification routes, the presence of nanoparticles normally brings additional benefits, including good mechanical performance, good conductivity, and excellent EMI shielding [25,26,27,28]. But there remains one main drawback of these nanoparticles, namely, they are not biodegradable. This produces environmental concerns regarding their fabrication, usage, and disposal. In this context, we recently proposed the use of cellulose nanofiber (CNF), which originates from the most abundant biopolymer on earth, as a cell nucleating agent to improve the foamability of iPP, and for the purpose of reinforcing the mechanical properties of PP foams [29,30,31]. As a natural and biodegradable material, CNF is a good alternative to conventional inorganic nanoparticles resulting from its biocompatibility, sustainability, renewability, and surface group functionality [32,33,34]. Moreover, CNF exhibits other important attributes including low density and good physical properties, such as high strength, good elasticity, and favorable thermal property [35,36]. However, very few studies have been conducted on fabricating composite foams containing CNF.

In our recent work [29,30,31], we have demonstrated the feasibility of preparing PP/CNF nanocomposite foams using FIM with core-back operation. Due to the hydrophilic nature of native CNF, it is difficult to disperse the unmodified CNF into hydrophobic polymers such as PP and PE, and poor adhesion is inevitably obtained at the interface of CNF and the hydrophobic polymer. To solve this issue, CNF was first modified using alkenyl succinic anhydride (ASA) and then compounded with iPP through a melting extrusion technology, which is eco-friendly and promising in large-scale processing for industrial application [29,30,31]. Very recently, the influence of modified CNF on the dispersion, rheological properties, and crystallization behavior of PP was comprehensively investigated, although the composite foams were only prepared at a fixed expansion ratio of 2-fold [37]. In this work, PP/CNF nanocomposite foams with different expansion ratios (2–10-fold) were prepared using the same core-back FIM technique. The effect of native and modified CNF on the cellular structures, tensile properties and compressive properties of PP foams were studied. 

## 2. Experimental Section

### 2.1. Materials

The PP used in this study was an iPP (grade F133A), which was supplied by the Prime Polymer Corp., Tokyo, Japan. It has a weight-average molecular mass of 379 kg/mol and displays a melt flow index of 3.0 g/10 min (2.16 kg load at 230 °C). Commercial nitrogen (N_2_) (99.9%, Izumi Sangyo, Kyoto, Japan) was used as the physical blowing agent for the foam injection molding experiments.

### 2.2. Preparation of PP/CNF Nanocomposites

Native and hydrophobic-modified CNF were prepared from needle-leaf bleached kraft pulp. Surface modification of the pulp was conducted prior to the fibrillation process. Briefly, the obtained pulp was surface-modified using alkenyl succinic anhydride (ASA), which was specified in our previous work [29,30]. The hydrophobicity of CNF was controlled by changing the ratio of cellulose to ASA. The degree of modification of the modified CNF was characterized by the degree of substitution (DS), which was determined using a Fourier transform infrared spectroscopy (FTIR) calibration curve [38]. Following our previous work [37], two different hydrophobic-modified CNFs, each with DS of 0.2 and 0.4, were prepared and used here. Then, the PP composites with native and modified CNF were prepared by melt-compounding the PP resin with cellulose using the following procedures: mixing, kneading, drying, and melt extruding in a twin-screw extruder, followed by pelletizing for the FIM. Finally, a 17 wt% PP/CNF master batch was prepared and used for the following injection molding experiments. 

### 2.3. Core-Back Foam Injection Molding Process

Foam injection molding (FIM) experiments were carried out using combination of a 35-ton clamping force injection mold machine (J35EL III-F, Japan Steel Work, Tokyo, Japan) and a Trexel gas dosing system (SCF device SII TRJ-10-A-MPD, Trexel Inc., Showa Tansan, Japan). The PP/CNF master batch was dry-blended with neat iPP, and an optimum concentration of 5 wt% CNF was used here, based on our earlier study [31]. For simplicity, PP/CNF composites with the unmodified CNF (DS = 0) and modified CNF with DS of 0.2 and 0.4 are referred to as CNF-0, CNF-0.2, and CNF-0.4, respectively. For comparison, pure iPP was also used for the foaming experiments. A rectangular mold with dimensions of 70 mm × 50 mm × 1 mm was used.

To produce foams with different expansion ratios, FIM with core-back operation was applied. The main difference between core-back FIM and the regular FIM process lies in an additional mold-opening operation. In the core-back FIM process, part of the mold can be quickly opened to expand the cavity volume, which simultaneously initiates the foaming process, caused by the rapid pressure drop, and produces a uniform cellular structure [7,8,9]. Thus, different expansion ratios of foams could be obtained by controlling the expanded cavity volume; detailed information on FIM with core-back operation was described previously [8,9]. Herein, N_2_ was used as the physical blowing agent and a 0.2 wt% N_2_ dosage was used. By fixing the core-back rate at 20 mm/s, core-back distance was changed to four values of 1, 4, 6, and 9 mm; thus, different expansion ratios of foams such as 2-, 5-, 7-, and 10-fold could be prepared. The optimum cellular structure was separately obtained and reported for each sample under the current processing condition. Other processing parameter details used during the core-back FIM experiments are given in Table 1.

### 2.4. Foam Morphology Characterization

To observe cell morphology, a tiny slice was cut from the middle of the injection-molded bars and cryogenically fractured after immersing in liquid nitrogen. Prior to observation, the prepared samples were gold-coated using a VPS-020 Quick Coater (Ulvac Kiko, Ltd., Miyazaki, Japan). Then, the microstructure was examined via a scanning electron microscope (Tiny-SEM Mighty-8, Technex Lab Co., Ltd., Tokyo, Japan).

Cell size was analyzed using ImageJ software and cell density, *N*_0_, was then calculated according to Equation (1):

(1) $$N_{0}={\left(\frac{n}{A}\right)}^{3/2}$$

where *n* is the number of cells in the selected micrographs and *A* is the area of the micrograph. 

### 2.5. Open Cell Content

The open cell content (OCC) of iPP and its composite foams were measured using a gas pycnometer (AccuPycII, Shimadzu, Kyoto, Japan) under a nitrogen environment. The measured volume value, *V_measure_*, from the gas pycnometer excluded the specimen’s open cell volume, and thus the *OCC* could be obtained using Equation (2):

(2) $$\mathrm{OCC}=\frac{V_{apparent}-V_{measure}}{V_{apparent}}\times 100\%$$

### 2.6. Thermal Analysis

Thermal behaviors of the injection-molded samples were investigated using a differential scanning calorimeter (DSC 7020, Hitachi High-Tech Science Corporation, Tokyo, Japan). Specimens of approximately 5–7 mg were cut from the middle of the solid and foamed injection-molded parts. Each sample was measured by heating from 30 to 200 °C at a heating rate of 10 °C/min under a nitrogen atmosphere.

### 2.7. Tensile Test

Tensile tests were conducted using a universal testing instrument (Autograph AGS-1 kN, Shimadzu, Japan) with a crosshead speed of 10 mm/min according to the ISO standard 37-4 at room temperature. Dog-bone shaped specimens were taken from the center of injection-molded bars and the prepared parts had a gauge length of 12 mm and width of 2 mm. The tensile properties were reported by averaging the results of at least five samples. Prior to mechanical testing, the foamed product was placed in an atmospheric environment to diffuse the gas for at least one month.

### 2.8. Compression Test

A universal testing instrument (Autograph AGS-1kN, Shimadzu, Japan) was used to investigate the compressive properties of foamed samples. Cubic specimens with a side length of 10 mm, cutting from the middle of injection-molded bars, were used for the compression tests. A crosshead speed of 1 mm/min was used and at least five specimens were measured for each condition at room temperature.

## 3. Results and Discussion

### 3.1. Evolution of Cell Morphology 

**Figure 1:**
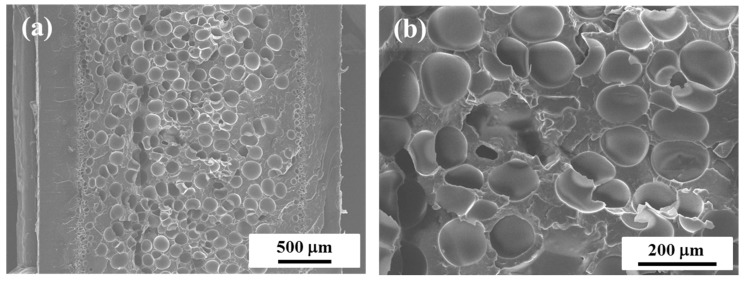
Typical SEM micrographs of: (**a**) the injection-molded isotactic polypropylene (iPP) foams; (**b**) the enlarged image of the core layer of (**a**).

Figure 1a shows the overall cell morphology of microcellular injection-molded iPP foams with a 2-fold expansion ratio. The SEM images display the microstructure of injection-molded foams from a view parallel to the core-back direction. It was observed that the injection-molded iPP foam exhibited a hierarchical morphology and could be divided into the solid layers (non-foamed layer) and foamed core layer. Figure 1b illustrates the magnified morphology of the core region of iPP foam, and we can observe that very large bubbles were generated in the iPP alone. This poor cellular structure was ascribed to the weak melt strength as well as poor crystallization behavior (formation of large crystals) of linear PP under the FIM processing condition. According to calculations, the cell density and averaged cell diameter for the 2-fold iPP foam were approximately 6.8 × 10^5^ cells/cm^3^ and 103 µm, respectively, which were congruent with our previous work [7,8,9]. 

**Figure 2:**
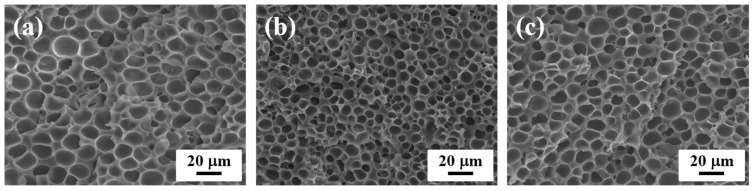
SEM micrographs of the cross-section of (**a**) cellulose nanofiber (CNF)-0, (**b**) CNF-0.2, and (**c**), CNF-0.4 foams at a fixed 2-fold expansion ratio.

Figure 2 displays the SEM images at the core region of the 2-fold PP/CNF composite foams with different DS. To better study the influence of CNF on the cellular structure of PP, in the following discussion, we mainly analyzed cell morphology in the core layer observed from the view perpendicular to the core-back direction [7,9]. Generally, compared with iPP foams, the added CNF greatly enhanced the cell nucleating ability of PP and produced finer foams with much smaller cell sizes and larger numbers of cells. In addition, with respect to the unmodified CNF, such improvement in cellular morphology was more conspicuous for composites with the modified CNF, which resulted from the promoted dispersion of modified CNF [37]. In our previous work [37], the X-ray CT results revealed massive bundles and very large agglomerations in the PP matrix reinforced with the native CNF, whereas much less agglomeration existed in the PP composites with the modified CNF. Moreover, they demonstrated that the cell structure of the CNF-0.2 sample was the finest among all the foams prepared at the 2-fold expansion ratio.

**Figure 3:**
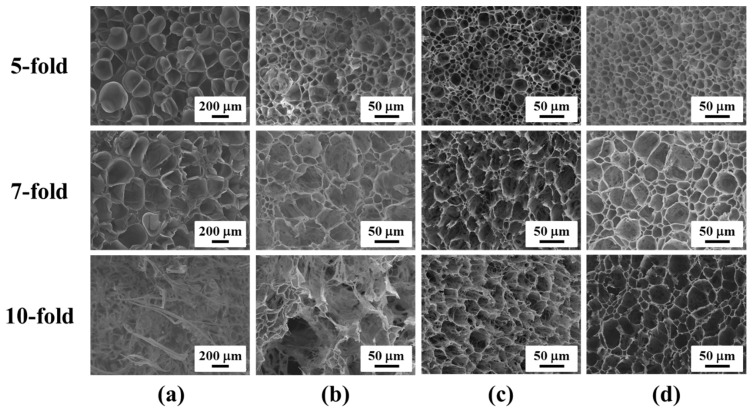
SEM micrographs of the cross-section of (**a**) iPP; (**b**) CNF-0; (**c**) CNF-0.2; and (**d**) CNF-0.4 foams in the core layer with different expansion ratios.

Figure 3 reveals the effect of native and modified CNF on the microstructure of PP foams with different expansion ratios. As noted previously, different expansion ratios of foams were prepared by changing the core-back distance. For example, 10-fold foam was obtained by enlarging the initial 1 mm mold-thickness to the final 10 mm through the core-back operation, while keeping the prepared foams well-expanded and integrated. Similarly, the cellular morphology was examined from the view perpendicular to the mold-opening direction. As shown in Figure 3a, large cells were inevitably generated for iPP foams with different expansion ratios. The presence of CNF clearly improved PP’s foam structures at all the investigated expansion ratios. Similar to the results at 2-fold, the hydrophobic-modified CNF revealed more enhancement in the foaming ability of PP than the native CNF. In addition, at the high expansion ratio of 10-fold, iPP foams could not maintain their spherical cell shape, even observed from the view perpendicular to the core-back direction, and displayed a fibrillary structure. This was due to the introduction of intensive elongation force during the core-back operation process, which would cause the deformation of the cell wall and subsequent cell coalescence and void formation [8,9,31]. Owing to the low melt strength and weak melt elasticity of iPP, its cell walls tended to rupture and break easily especially at high expansion ratios. In contrast, spherical cell shapes were maintained for the PP/CNF composite foams at 10-fold, revealing the role of CNF in stabilizing the cellular structure of PP. This was achieved by the promoted crystallization of PP as well as the increase in its melt strength with the addition of CNF [29,37].

### 3.2. Analysis of the Cellular Parameter 

**Figure 4:**
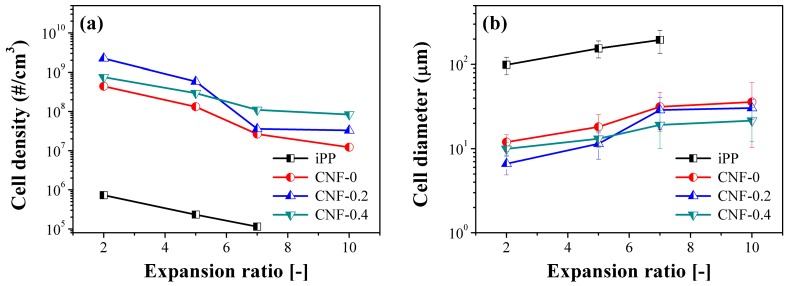
(**a**) Cell density and (**b**) average cell diameter of the neat iPP and polypropylene (PP)/CNF nanocomposite foams as a function of the expansion ratio.

Figure 4 summarizes the cell density and average cell diameter variables for iPP and PP/CNF composite foams as a function of the expansion ratio. It was revealed that iPP foams always possessed a low cell density of approximately 10^5~6^ cells/cm^3^ at different expansion ratios. With the presence of CNF, cell densities of the PP foams were greatly increased to 10^7~9^ cells/cm^3^, realizing an increase of 2–3 orders of magnitude for the different expansion ratios of foams. Such substantial improvements in the cell density for PP foams were ascribed to the addition of CNF. In our previous reports [29,37], it was clearly demonstrated that the added CNF could act as the crystal nucleating agent for PP and enhanced the crystallization rate, resulting in the formation of large quantities of small-sized crystals. These changes in the crystallization property of PP would greatly affect its final foaming behavior. According to the literature [39,40,41], the formation of plenty of tiny crystals in semi-crystalline polymer could provide more cell nucleating sites and increase local gas supersaturation, and thus, the promoted crystallization in PP/CNF composites would contribute to their formation of fine cellular structures with much smaller cell sizes [7,40,42]. Correspondingly, the nano-sized CNF could equally play the role of bubble nucleating agent and improve the foaming ability of PP. Moreover, the added CNF enhanced the melt strength of PP and this would be beneficial for the restriction of cell growth, together with diminishing cell coalescence [7,37].

As shown in Figure 4, if we go into more detail, the native and modified CNF had different promoting effects in the cellular parameters of PP and these were closely related to the detailed values of the expansion ratios. At the low expansion ratio of 2- and 5-fold, the CNF-0.2 sample exhibited the highest cell density and smallest cell sizes, followed by the CNF-0.4 sample. For example, cell density for the iPP, CNF-0, CNF-0.2, and CNF-0.4 foams at 5-fold were 2.33 × 10^5^, 1.32 × 10^8^, 5.82 × 10^8^, and 2.95 × 10^8^ cells/cm^3^, respectively. In summary, at low expansion ratios of 2- and 5-fold, changes in cell density followed the sequence of CNF-0.2 > CNF-0.4 > CNF-0 > iPP. Our previous crystallization results revealed that the variation of the crystallization rate was CNF-0.2 > CNF-0.4 > CNF-0 > iPP, which was the same as the changing cell density for low expansion-ratio foams. Generally, the role of nanoparticles in promoting the foaming ability of polymer could be classified into the melt-strength promoted factor and the crystallization promoted factor. Given that these two factors always combine, it is rather difficult to state which one is dominant. Herein, by changing the expansion ratio, we wanted to uncover the factors controlling or dominating the expansion ratio. Moreover, our earlier findings indicated that CNF-0.4 had the highest melt viscosity and melt elasticity, and even that a network was generated within the composite, which was ascribed to the good dispersion of modified CNF and its long fibrillar structure [37]. This was followed by the similar melt properties of CNF-0.2 and CNF-0 composites, while iPP exhibited the lowest melt viscosity and melt elasticity. Considering the changes in crystallization behaviors and melt properties, it is reasonable to say that the crystallization-promotion-effect dominates the foaming behavior of iPP and PP/CNF nanocomposite at the 2- and 5-fold expansion ratios. This can be understood as follows: the pre-existing crystals could act as the cell nucleation sites and enhance the cell nucleation process, and hence promote the foaming property of the polymer. Since the CNF-0.2 sample had the fastest crystallization rate and the possible formation of the largest amounts of initial tiny crystals at high temperature, this would supply more nucleating sites, and thus obviously improve the foaming behavior and produce the finest cellular structure in CNF-0.2 sample.

In contrast, at the high 7- and 10-fold expansion ratios, CNF-0.4 exhibited the finest cellular structures, and in terms of cell density, CNF-0.4 > CNF-0.2 > CNF-0 > iPP. For instance, at the expansion ratio of 7-fold, cell density of the neat iPP, CNF-0, CNF-0.2, and CNF-0.4 foams was 1.15 × 10^5^, 2.64 × 10^7^, 3.59 × 10^7^, and 1.10 × 10^8^ cells/cm^3^, respectively. This indicated that the crystallization-promotion factor was not the main cause of the foaming behavior at a high expansion ratio. Unlike the CNF-0.2 and CNF-0 samples, a rheological network was generated in the CNF-0.4 specimen [37]. The formation of a network structure in the CNF-0.4 sample was beneficial for the increasing of melt strength, which was helpful in suppressing bubble breakage and bubble coalescence. This was extremely important for the high expansion-ratio injection-molded foams, as the high extensional force was always concurrent, and it would significantly stretch the cell wall and affect the cell growth stage [30,31]. Moreover, to explore the effect of CNF on the foamability of PP, we tested the maximum expansion ratio for each material in the FIM experiments. It was found that the maximum expansion ratios for iPP, CNF-0, CNF-0.2, and CNF-0.4 foams were 10-, 13-, 17-, and 20-fold, respectively. Due to the low melt strength and weak elasticity, iPP foams could easily collapse at high expansion ratios and could not maintain their integrated cellular structures. In our previous work [30], it was revealed that the maximum expansion ratio was about 12-fold for the long-chain branching PP, with obvious strain-hardening behavior. Thus, the highest expansion ratio achieved in the CNF-0.4 sample was possibly related to the formation of a network structure, which would be the key to keeping the overall cellular skeleton and avoiding serious cell rupture caused by the intensive extensional force induced during the core-back operation process.

**Figure 5:**
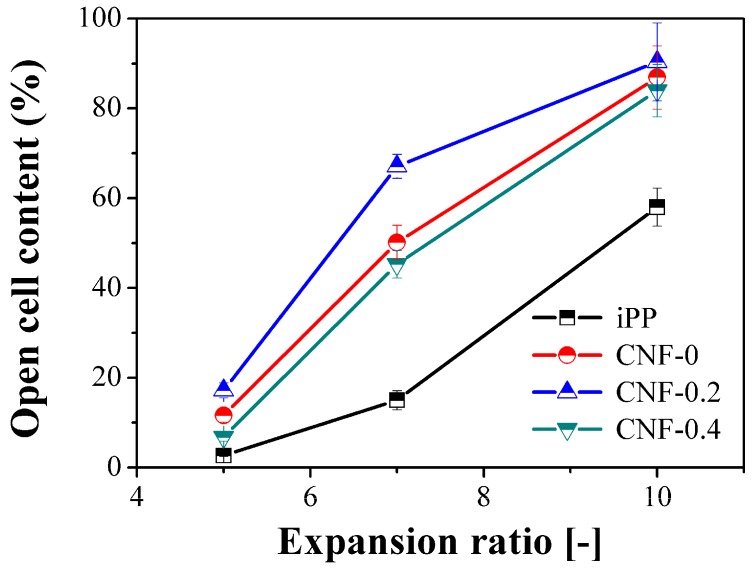
Open cell content for iPP and PP/CNF nanocomposite foams as a function of the expansion ratio.

This can also be validated from the variation in the degree of opening of the cell in different samples. Figure 5 shows the effect of the expansion ratio on the open cell content (OCC) for iPP and PP/CNF composites. Compared with the iPP foams, adding both the native and modified CNF increased the OCC of PP foams, which was ascribed to the promoted cell nucleation caused by the enhanced crystallization and concurrent thinner cell wall [37,40]. In addition, the changing sequence of OCC was the same as the order of crystallization rate for iPP and PP/CNF composites [37]. Taking 7-fold foams as an example, the OCC for iPP, CNF-0, CNF-0.2, and CNF-0.4 foams was about 15%, 50%, 67%, and 45%, respectively, revealing that the added CNF could not only act as the cell nucleating agent, but also as the bubble opening agent for PP. Compared with the CNF-0 and CNF-0.2 samples, a relatively low OCC was achieved in the PP/CNF composite foams with the DS of 0.4. This can provide indirect evidence of the role of the formation of a network structure in stabilizing the cellular structure and diminishing cell breakage. In summary, the crystallization-promotion factor was dominant for preparing low expansion-ratio injection-molded foams since the cell nucleation process prevails over the foaming process, while the melt-strength-promotion factor dominates the production process for high expansion-ratio foams, whereas the foaming process is more related to the cell growth process and mold-opening operation.

### 3.3. Tensile Results 

**Figure 6:**
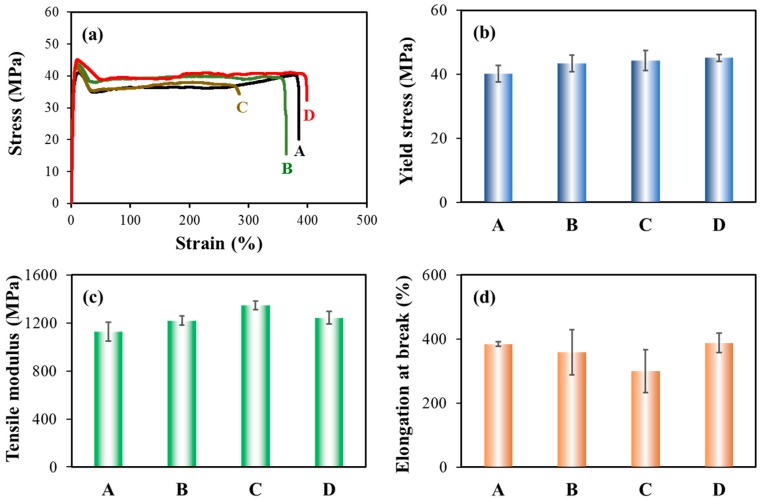
Tensile results of the solid injection-molded parts: (**a**) typical stress–strain curves; (**b**) yield stress; (**c**) tensile modulus; and (**d**) elongation at break of (A) iPP, (B) CNF-0, (C) CNF-0.2 and (D) CNF-0.4 specimens.

The mechanical properties of the neat iPP and PP/CNF composites were studied by the tensile tests. Firstly, we investigated the effect of CNF on the mechanical performance of the solid injection-molded PP. Figure 6 shows the obtained stress–strain curves, yield stress, tensile modulus, and elongation at break for the solid injection-molded products. As expected, pure iPP exhibited an obvious necking phenomenon and a ductile failure as shown in Figure 6a. A similar mode of ductile failure was observed for the PP/CNF nanocomposites. As for the solid iPP, the yield stress and tensile modulus were 40.2 MPa and 1126.9 MPa, respectively. The addition of native CNF to PP enhanced the yield stress and the tensile modulus of PP to 43.4 MPa and 1219.7 MPa, respectively. This reinforcement effect resulted from the well-known reinforcing mechanisms of stiff nanoparticles in a soft matrix, since the added fibers can support the stresses transferred from the polymer [43,44]. In contrast, the PP composites containing the modified CNF at a DS of 0.2, displayed a yield stress and tensile modulus of 44.3 MPa and 1346.9 MPa, respectively, which were about 10% and 20% higher than those of the iPP product. Similarly, the mechanical response of the CNF-0.4 sample was enhanced by 12% and 10% for yield stress and tensile modulus, respectively. Compared with the native CNF, a higher reinforcing efficiency was observed in PP composites with modified CNF. This greater improvement was attributed to the good interaction between modified CNF and PP, possibly resulting from the incorporation of the double alky chain structure of ASA into the surface of CNF [36,37]. Correspondingly, the elongation at break of PP was decreased by the incorporation of CNF, which was a typical behavior for the polymer composite with an added particle [43]. This decrease was more obvious for the CNF-0.2 sample, which reduced the elongation at break from 384% for iPP to 300% for the composites. This was similar to other systems of polymer mixed with filler, which was due to the decreasing deformability of a rigid interphase between the polymer resin and the stiff filler [36,43,44,45].

**Figure 7:**
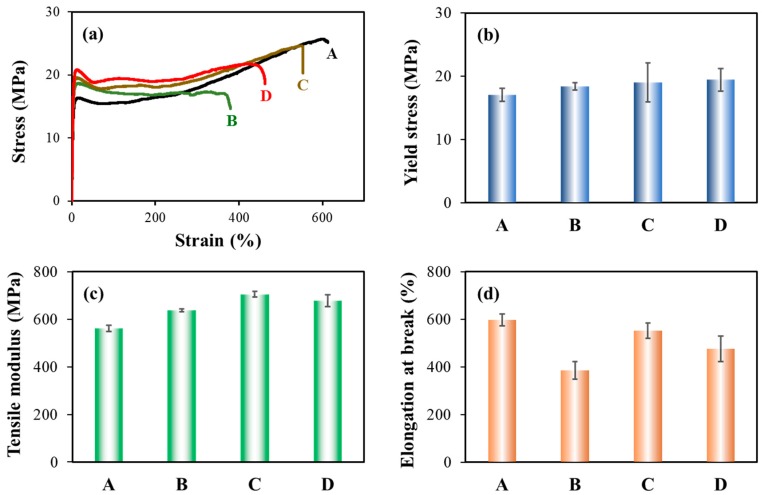
Tensile properties of the injection-molded foams with a 2-fold expansion ratio: (**a**) typical stress–strain curves; (**b**) yield stress; (**c**) elongation at break; and (**d**) tensile modulus of (A) iPP, (B) CNF-0, (C) CNF-0.2 and (D) CNF-0.4 specimens.

Moreover, we studied the tensile properties of foamed sample at the low expansion ratio of 2-fold, whereas other expansion-ratio foams were inappropriate to test due to their large thickness. Figure 7 illustrates the typical stress-strain curves, yield stress, elongation at break, and tensile modulus for the foamed iPP and PP/CNF nanocomposites. The foamed samples displayed a similar tensile behavior as the solid injection-molded bars, fracturing in a ductile manner with obvious yield and necking. Generally, the mechanical strength of all the samples was decreased by foaming, which was due to incorporation of the void fraction. For instance, the yield stress and tensile modulus were reduced from 40.2 MPa and 1126.9 MPa for solid iPP, to 17.1 MPa and 561.5 MPa for 2-fold iPP foams. The addition of native and modified CNF again reinforced the mechanical strength of PP foams and similarly, more improvement was observed in PP composites containing modified CNF. Compared with the iPP foams, the yield stress of the CNF-0, CNF-0.2 and CNF-0.4 composites was enhanced by 8%, 12%, and 14%, respectively. This percent increment was similar to that of the solid sample. However, a more significant increase was achieved in the tensile modulus. Specifically, tensile moduli of the CNF-0, CNF-0.2 and CNF-0.4 composites were enhanced by 14%, 26%, and 21%, respectively, with respect to the iPP foam; while the corresponding values were about 8%, 20%, and 10%, respectively, for the solid injection-molded iPP product. This demonstrated that the added CNF was more effective in reinforcing the mechanical strength of foams in comparison to its solid counterpart, which was attributed to improved stress transfer due to incorporating CNF together with the improved cellular structures.

**Figure 8:**
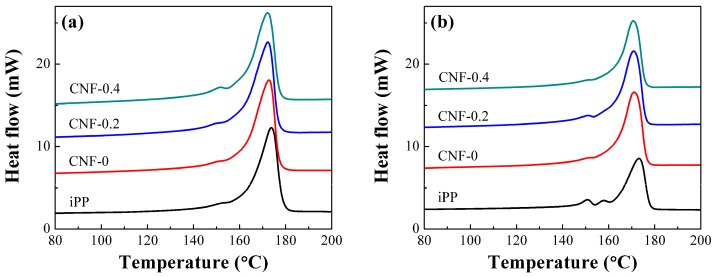
The first heating curves of the core region for: (**a**) solid, and (**b**), 2-fold foamed iPP and PP/CNF nanocomposites.

As displayed in Figure 7d, the elongation at break of iPP foam was greatly increased compared to its solid injection-molded product, exhibiting an approximately 55% increment. This obvious increase in elongation at break was unusual for PP foams when considering their relatively large cell sizes (about 100 μm). To explore the factors increasing the elongation at break of PP, we studied the crystal structures of the samples by differential scanning calorimeter (DSC). Figure 8 displays the melting curves in core region of the iPP and the PP/CNF nanocomposites. Apart from the main melting peak at around 173 °C, a weak melting peak at around 146–152 °C was observed for the solid iPP (Figure 8a). This lower melting peak was ascribed to the formation of β-crystal in iPP, which was sometimes found in the injection molding process of PP [46]. As shown in Figure 8, most of the samples exhibited a very weak peak of β-crystal, signifying very low content of β-crystal. In contrast, a more obvious melting peak for β-crystal was observed for the 2-fold iPP foams. This revealed that a relatively high content of β-crystal was generated in the core area of the iPP foam, which was also confirmed by our previous results [37]. This resulted from the introduction of extensional force during the core-back operation, since α-nuclei of PP was prone to induce the formation of β-nuclei on its surface under an appropriate flow field [47,48,49]. It is known that α-crystal of PP exhibits greater strength than that of β-crystal, while a higher ductility is achieved in β-crystals compared to the α-crystal of PP [48,50]. By calculating [46], the content of β-crystals was increased from approximately 3.4% for the solid iPP to 15.1% for the 2-fold iPP foams. Thus, it is reasonable to say that the higher elongation at break in iPP foams relative to its solid counterpart is mainly attributed to the formation of more β-crystal in iPP. Moreover, elongation at break of the PP/CNF nanocomposite foams was also improved after foaming. The elongation at break of CNF-0, CNF-0.2 and CNF-0.4 composites was enhanced by 8%, 85%, and 23%, respectively, with respect to their solid counterparts. Since the added CNF worked against the formation of β-crystals [37], the increase in elongation at break for the PP/CNF nanocomposite foams was mainly due to the efficiency of transferring applied stress enhanced by the incorporation of microcellular cells. From the above SEM results, it was found that the CNF-0.2 sample had the smallest cell size (approximately 6.6 μm) and highest cell density (4.48 × 10^9^ cells/cm^3^). This finest cellular structure gave the CNF-0.2 sample with the largest increase in elongation at break. Compared with other composite foams, the cell walls were thinner in the CNF-0.2 sample due to the smaller cell sizes and higher cell densities, and thus these struts and cell walls were more easily deformed. Under tensile testing, highly fibrillated cells were produced along the drawing direction by interconnecting with the nearby microscale cells, which were beneficial for shear yielding of piles of fibrils along the stretching direction. Therefore, much higher elongation at break was achieved in the CNF-0.2 composite foams, which was similar to our previous work on long-chain branching PP (LCBPP) with an added nucleating agent.

### 3.4. Compression Results 

**Figure 9:**
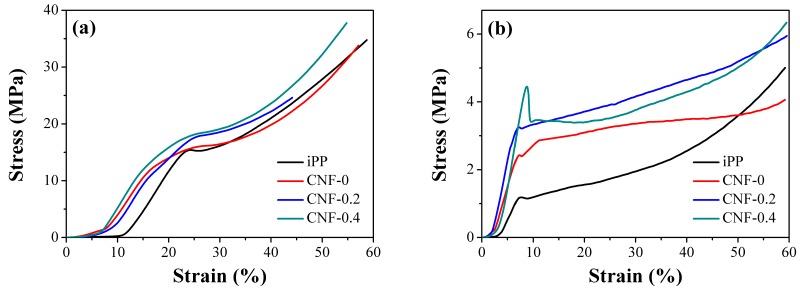

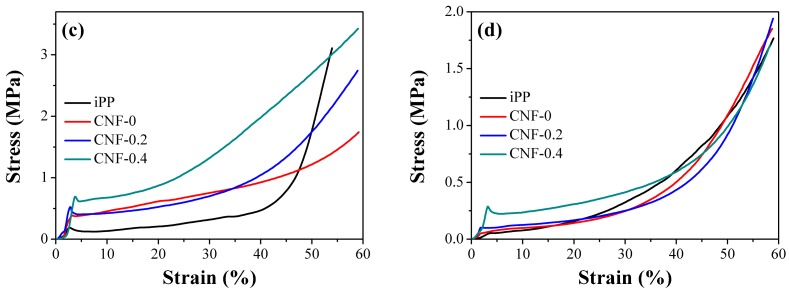
Characteristic stress–strain curves for foamed iPP and PP/CNF nanocomposites at the expansion ratios of: (**a**) 2-fold; (**b**) 5-fold; (**c**) 7-fold; and (**d**) 10-fold, respectively.

Figure 9 shows the typical compressive stress–strain curves for different expansion ratios of iPP and PP/CNF composite foams. As can be observed, regardless of the type of CNF, the mechanical performance of the PP/CNF composites was much higher than that of the iPP foams, which indicates the further reinforcing effect of the added CNF on high expansion-ratio PP foams. Figure 9a shows that stress increased with enhanced strain for all 2-fold foams, which was due to the formation of closed cells in such a low expansion ratio. As for the closed-cell foam, the compression of gas within the cells, as well as the membrane stress that occurred in the cell faces, typically led to a stress increase with strain [51]. In contrast, other expansion ratios of foamed samples exhibited similar stress–strain behaviors as the elastomeric open-cell foams (Figure 9b–d), which were characterized by three distinct stages: a linear elastic stage, a collapse plateau stage and a densification stage [51,52]. 

**Figure 10:**
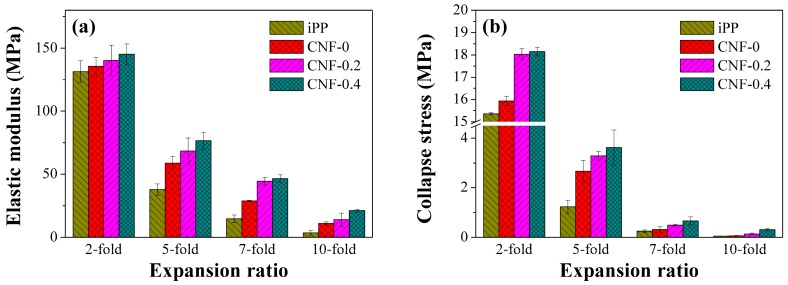
Change of (**a**) elastic modulus and (**b**) collapse stress for the iPP and PP/CNF nanocomposite foams as a function of the expansion ratio.

**Figure 11:**
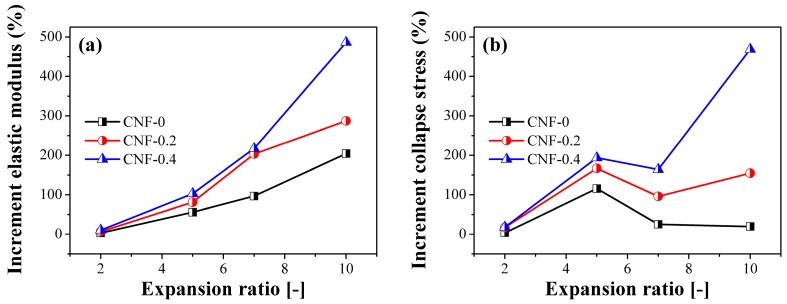
Percentage increment in mechanical response for the PP/CNF nanocomposite foams: (**a**) increments in elastic modulus, and (**b**) increments in collapse stress as a function of the expansion ratio.

To understand the role of CNF, the elastic modulus and collapse stress were calculated for the iPP and PP/CNF composite foams. The elastic modulus was obtained from the slope of the initial linear elastic region of the stress–strain curve. Figure 10 shows the experimental results of elastic modulus and collapse stress of iPP and those of composite foams. As expected, the elastic modulus of all the foamed specimens decreased with an increase of the expansion ratio (Figure 10a), due to the density reduction and the larger cellular sizes. It also shows that the added CNF improved the elastic modulus of PP foams. The elastic modulus of 2-fold iPP, CNF-0, CNF-0.2, and CNF-0.4 foams were 131.4, 135.7, 149.4, and 145.3 MPa, respectively, which indicates a slight increase of elastic modulus at a low expansion ratio. Furthermore, comparing with the effect of CNF, the percentage increment of the composite foams with respect to the iPP was calculated and is shown in Figure 11. As shown in Figure 11a, compared with iPP, the percentage increment in the elastic modulus of CNF-0, CNF-0.2 and CNF-0.4 composites at the 5-fold expansion ratio were 56%, 81%, and 103%, respectively. As presented, the modified CNF always exhibited a higher elastic modulus than the native CNF, realizing our aim of improving the mechanical properties of PP with hydrophobic-modified CNF. In addition, among all the samples, CNF-0.4 had the highest elastic modulus and provided the strongest reinforcement effect for PP foams. This can be explained by the open cell content exhibited by the PP/CNF composites. It is known that the formation of opening cells has a negative impact on the mechanical performance of foams [51]. From the above SEM results, it was shown that the CNF-0.4 sample had the lowest open cell content, and thus the highest elastic modulus was achieved in the CNF-0.4 composite. Moreover, the added CNF had the highest percentage increase in elastic modulus for the 10-fold foam, reaching 204%, 288%, and 486% for CNF-0, CNF-0.2 and CNF-0.4, respectively. This clearly signified that the presence of CNF was extremely effective in reinforcing the mechanical properties of the high expansion-ratio foams.

The experimental results of collapse stress for the iPP and PP/CNF composites are plotted along the expansion ratio in Figure 10b. An increment in collapse stress was observed for PP based composites due to the addition of CNF. Due to density reduction, high expansion-ratio foams always exhibited lower values of collapse stress than the low expansion-ratio foams. Even though iPP foams exhibited relatively low open cell contents, the addition of CNF invariably strengthened the collapse stress of PP and this reinforcement effect was more obvious for the modified CNF. This improvement in the foamed nanocomposites lies in two main reasons: firstly, the added nanoparticles can improve both the stiffness and strength of the polymeric matrix comprising cell walls of the foams; and second, the CNF plays the role of a cell nucleating agent, which is favorable for increasing the cell density and decreasing the cell size, together with improving the homogeneity of the cell size distribution. 

A similar analysis of elastic modulus was carried out for collapse stress and percentage increment in terms of collapse stress for PP/CNF composites with respect to iPP and the results obtained are shown in Figure 11b. It was found that the percentage increase in collapse stress for PP/CNF nanocomposite foams first increased for 2-fold to 5-fold samples and then decreased at the 7-fold expansion ratio. The relatively lower increment in collapse stress for the 7-fold PP/CNF nanocomposite foams was due to the large difference in open cell content between the neat iPP and PP/CNF composite foams. Specifically, the open cell content for the 7-fold iPP, CNF-0, CNF-0.2, and CNF-0.4 foams was 15%, 50%, 67%, and 45%, respectively, and these large amounts of opening cells in the PP/CNF composites would partially counteract their mechanical strengthening of PP. Finally, the percentage increase in collapse stress again increased for the 7-fold to 10-fold PP/CNF composite foams. This was attributed to the reinforcement effect of the stiff CNF and the concurrent improvement in cellular structures, while the above SEM results revealed that markedly poor cellular morphologies were observed in the 10-fold iPP foams. For instance, a very high percentage increment in collapse stress of approximately 469% was achieved in the 10-fold CNF-0.4 sample, while the corresponding values were approximately 20% and 155% for the CNF-0 and CNF-0.2 composites. These findings clearly demonstrate that the addition of hydrophobic-modified CNF is a viable approach to refine the foaming ability of PP for preparing high expansion-ratio injection-molded foams as well as reinforcing their mechanical performance, which potentially enlarges the application of lightweight PP foams into areas such as construction and transportation.

## 4. Conclusions

PP/CNF nanocomposite foams incorporating native and modified CNF with different expansion ratios were prepared via a core-back FIM process. It was revealed that the addition of CNF greatly improved the cellular structures of PP, and the PP/CNF (DS = 0.2) composite exhibited the smallest cell sizes and highest cell densities for its 2- and 5-fold foams. This was ascribed to the crystallization-promotion-effect that dominated the foaming behavior of low expansion-ratio foams, with the PP/CNF (DS = 0.2) composite the one possessing the best crystallization property. In contrast, for high expansion-ratio foams including 7- and 10-fold, the finest cellular structures were achieved in the PP/CNF (DS = 0.4) specimen. Such improvement in cellular structure resulted from the melt-strength dominating effect for high expansion-ratio injection-molded foams. Since a rheological network was generated in PP/CNF (DS = 0.4), it not only endowed CNF-0.4 with finest cellular structures at high expansion ratios but also produced the maximum expansion ratio foams. Additionally, the open cell content results provided further evidence for the above findings. It was demonstrated that CNF-0.2 had the largest OCC, due to the thinner cell wall caused by enhanced crystallization and consequent increased cell nucleation; while the high melt strength of CNF-0.4 gave it a relatively low OCC. Accordingly, the addition of CNF brought out an increase in the tensile modulus and yield stress of both the solid and foamed PP samples. Compressive results also validated the finding that the presence of native and modified CNF improved the mechanical properties of PP foams, and that more improvement was achieved in the PP composites with modified CNF. As for expansion ratios as high as 10-fold, the percentage increments in the elastic modulus for CNF-0, CNF-0.2, and CNF-0.4 were 204%, 288%, and 486%, respectively, with respect to iPP foam. Moreover, the CNF-0.4 sample exhibited a notable increase in the collapse stress, reaching approximately 5.7 times higher than that of the 10-fold iPP foam. The present work reveals that the incorporation of CNF is a feasible method to develop high expansion-ratio PP composite foams with fine cellular structures and good mechanical properties, which can possibly be applied in the construction and transportation industries. 

## Figures and Tables

**Table 1:** Processing conditions for the core-back foam injection molding experiments.

Parameters	Values
Barrel temperature (°C)	180, 200, 230, 220, 210, 210, 210
Mold temperature (°C)	40
Injection speed (mm/s)	70
Injection pressure (MPa)	180
Shot size (mm)	35
Packing pressure (MPa)	60
Dwelling time (s)	2.0–4.0
Core-back distance (mm)	1–9
Core-back rate (mm/s)	20
N_2_ content (wt %)	0.2
